# Experiences of Red River Métis Accessing COVID Vaccines: A partnership-based, whole-population linked administrative data study.

**DOI:** 10.23889/ijpds.v7i3.1944

**Published:** 2022-08-25

**Authors:** Nathan C. Nickel, Julianne Sanguins, Okechukwu Ekuma, Carole Taylor, Nkiruka Eze, Oludolapo Deborah Balogun, Hera Casidsid, Marni Brownell, Mariette Chartier, Francis Chartrand, Daniel Chateau, Michelle Driedger, Jennifer Enns, Marni Brownell, Alan Katz, Olena Klos, Lisa Lix, Alyson Mahar, Rachelle Neault, Marcelo Urquia

**Affiliations:** 1 Manitoba Centre for Health Policy, University of Manitoba; 2 Manitoba Metis Federation (MMF); 3 University of Manitoba; 4 Australian National University

## Objectives

Red River Métis are Indigenous people hailing from the Canadian Prairies who have historically experienced poor health outcomes due to colonial practices. Researchers from the Manitoba Métis Federation (MMF) partnered with health services researchers to test whether MMF-led COVID initiatives were associated with access to COVID-19 testing and vaccines.

## Approach

We linked the Métis Population Data-Base from the MMF (to identify Red River Métis) with whole-population COVID testing and vaccination data and health and social services administrative data (for information on sociodemographics and confounders) to complete this retrospective cohort study. We used restricted mean survival time models to test whether COVID-19 vaccination differed between Métis and all other Manitobans (AOM); models adjusted for demographics, comorbidities, and other characteristics (age, socioeconomic status, urbanicity, and mental health status). Data were stratified by sex and subsequent effect modification analyses tested whether associations differed by sex and physical health comorbidities.

## Results

COVID testing rates were lower during the first year of the pandemic among Métis than among AOM. During the second year of the pandemic, this finding was reversed - Métis accessed tests at higher rates. There was no difference between Métis and AOM in accessing first vaccine doses before implementation of MMF-led initiatives. After initiatives were put in place, Métis received their second COVID vaccine, on average, 1.3 (95% CI 1.9-0.6) days sooner than AOM, after adjusting for confounders. Effect modification analyses showed this relationship was concentrated among females – female Métis received their second vaccine 1.7 (2.6-0.8) days sooner than female AOM; differences were non-significant for males. Métis with 2+ comorbidities received their vaccine second 2.9 (5.3-0.5) days sooner than AOM with 2+ comorbidities.

## Conclusion

Public health initiatives prioritizing Métis for vaccines improved uptake. Initiatives led by Métis to improve COVID outcomes were critical to supporting Métis during the course of the pandemic. Public health response efforts need to operate from a standpoint that honours Indigenous sovereignty in their design and implementation.

